# Prognostic Significance of Serum Uric Acid and Exercise Capacity in Older Adults Hospitalized for Worsening Cardiovascular Disease

**DOI:** 10.3390/jcdd11060165

**Published:** 2024-05-26

**Authors:** Akihiro Hirashiki, Atsuya Shimizu, Takahiro Kamihara, Manabu Kokubo, Kakeru Hashimoto, Ikue Ueda, Toyoaki Murohara

**Affiliations:** 1 Department of Cardiology, National Center for Geriatrics and Gerontology, Obu 474-8511, Japan; ashimizu@ncgg.go.jp (A.S.); kamihara@ncgg.go.jp (T.K.); mkokubo@ncgg.go.jp (M.K.); 2 Department of Cardiology, Nagoya University Graduate School of Medicine, Nagoya 466-8560, Japan; murohara@med.nagoya-u.ac.jp; 3 Department of Rehabilitation, National Center for Geriatrics and Gerontology, Obu 474-8511, Japan; khashi@ncgg.go.jp (K.H.); ikueueda@ncgg.go.jp (I.U.)

**Keywords:** uric acid, older adults, exercise capacity, prognosis

## Abstract

Elevated serum uric acid (sUA) is associated with the risk of developing cardiovascular disease (CVD). Here, we examined the prognostic significance of sUA and exercise capacity in 411 Japanese adults (age, ≥65; mean, 81 years) hospitalized for worsening CVD. When the patients were stratified by sUA into three groups (<5.3, 5.4–6.9, >7.0 mg/dL), the high-sUA group had a significantly worse peak VO_2_ and composite endpoint (rehospitalization due to worsening CVD and all-cause mortality) compared with low- and moderate-sUA groups (*p* < 0.001). When the patients were stratified by sUA into five groups (sUA < 3.9, 4.0–5.9, 6.0–7.9, 8.0–8.9, and >10.0 mg/dL), the incidence of the composite endpoint was significantly higher in the highest sUA group compared with that in the reference group, but only in women. Univariate Cox regression analysis, but not a multivariate analysis, indicated that sUA was significantly associated with the composite endpoint. Although sUA and exercise capacity may have some degree of prognostic significance in older patients with CVD, this significance may differ between men and women.

## 1. Introduction

Uric acid (UA) is a waste product of purine metabolism and a mediator of several pathological processes, including oxidative stress, inflammation, and endothelial dysfunction [1,2]. Decreased excretion and increased synthesis of UA both contribute to higher circulating UA concentrations. Hyperuricemia is a risk factor for, and marker of, cardiovascular disease (CVD) [3,4,5]. It is also associated with the incidence and prognosis of heart failure (HF) [6]. Epidemiologic, experimental, and clinical data show that patients with hyperuricemia are at increased risk of cardiac, renal, and vascular damage, and cardiovascular (CV) events [7,8,9]. 

The association between UA and CV events was first reported by Kannel et al. in 1967 [10]; they showed that subjects with hyperuricemia had an increased risk of myocardial infarction. More recently, evidence was found of an association between hyperuricemia and chronic metabolic syndrome [1], kidney disease [11], hypertension [12], diabetes mellitus [13], and acute coronary syndrome [14]. More detailed information regarding the predisposition of individuals with hyperuricemia to oxidative stress and its effects needs to be elucidated in order to initiate the appropriate treatment for the optimal maintenance of UA levels, thereby improving the long-term prognosis and quality of life of patients [1]. 

CV risk factors and outcomes differ between men and women [15]. Gender differences are also apparent in HF patients with regard to etiology, left ventricular ejection fraction (LVEF), and prognosis [16,17]. The association between sUA and CV disease outcomes appears to be more pronounced in female patients than in male patients [18,19] but the role of gender in the relationship between sUA and survival in HF patients has not been clearly established.

The pathophysiology of older adults with CVD is complex and involves multiple dysregulated pathways. Potentially, general laboratory parameters such as sUA and exercise capacity may be useful as biomarkers for assessing prognosis in older adults with CVD. However, although the association between elevated sUA, CVD, and mortality is well recognized [1,2], it is still undecided whether the association reflects a causal inference or whether sUA is a risk marker reflecting the burden of the underlying disease. In addition, there was little information on the association between sUA and exercise capacity in older adults with CVD. Therefore, here, we examined the prognostic significance of sUA and exercise capacity in older Japanese adults with CVD.

## 2. Materials and Methods

### 2.1. Study Population

A total of 411 of patients aged ≥65 years who were hospitalized for CVD in the Department of Cardiology at the National Center for Geriatrics and Gerontology, Obu, Japan, between August 2018 and March 2023 were enrolled in the study. All of the patients were enrolled once they were stable and on optimal pharmacological therapy according to current guidelines for the treatment of CVD. The inclusion criteria were structural heart disease consisting of coronary artery disease (having experienced angina pectoris or myocardial infarction, with or without a history of revascularization procedures), symptomatic HF (including conditions such as non-ischemic cardiomyopathy, ischemia, tachycardia, bradycardia, valvular disease, and hypertension), and aortic disease, peripheral artery disease, and other vascular diseases. Non-ischemic cardiomyopathies were considered as ventricular myocardial abnormalities in the absence of coronary artery disease or valvular, pericardial, or congenital heart disease. Tachycardia and bradycardia included atrial, supraventricular, and ventricular arrhythmias, sick sinus syndrome, and atrioventricular block in the absence of structural heart disease. Valvular heart disease was diagnosed on the basis of hemodynamic or echocardiographic findings or a history of valvular or congenital cardiac surgery. Hypertension was defined as systolic blood pressure ≥ 140 mmHg, diastolic blood pressure ≥ 90 mm Hg, or a history of treatment for hypertension. HF was defined as pulmonary venous congestion or edema on chest X-ray plus any indicative symptoms (e.g., dyspnea, ankle swelling, peripheral edema, or fatigue).

The exclusion criteria were severe respiratory dysfunction (receipt of long-term oxygen therapy for respiratory disease), liver dysfunction (Child–Pugh score class C), stroke, renal dysfunction (albuminuria and glomerular filtration rate category G5), malignant tumors carrying a prognosis of less than 1 year, difficulty walking 10 m even with a walking aid, Mini-Mental State Examination score less than 18 points, and living in a nursing care facility before admission.

### 2.2. Study Overview

Within three days of study enrollment, a standard physical examination was conducted and standard laboratory, echocardiography, physical function, and cardiopulmonary exercise test (CPX) parameters were evaluated in the patients. All of the patients were in a stable condition at the time of testing. We then retrospectively examined the influence of sUA on prognosis using a composite endpoint of rehospitalization due to worsening HF and all-cause mortality. The study protocol complied with the Declaration of Helsinki, and written informed consent was obtained from each subject. The ethics review board of the National Center for Geriatric and Gerontology approved the study (approval no. 1272).

### 2.3. CPX Procedure

Each patient underwent CPX on a cycle ergometer at a progressively increasing work rate up to maximum tolerance. The test protocol was conducted in accordance with the recommendations of the American Thoracic Society and American College of Chest Physicians [20]. The oxygen and carbon dioxide sensors were calibrated before each test with known oxygen, nitrogen, and carbon dioxide concentrations. Test termination criteria were patient request, volitional fatigue, ventricular tachycardia, or ≥2 mm horizontal or downsloping ST-segment depression during exercise. A qualified exercise physiologist conducted each test under a physician’s supervision. A 12-lead electrocardiogram was monitored continuously, and blood pressure was measured every minute during exercise and throughout the 5 min recovery period. Respiratory gas exchange variables, including VO_2_, VCO_2_, and minute ventilation (VE), were acquired continuously throughout the test with an Oxycon Pro ergospirometer (CareFusion; San Diego, CA, USA); percent gas-exchange data were obtained breath-by-breath. Peak VO_2_ and peak respiratory exchange ratio were determined as the highest 30 s average values obtained during the final stage of the test. The ratio of the increase in VO_2_ to the increase in work rate (WR) [ΔO_2_/ΔWR] was calculated by least-squares linear regression from the data recorded between 30 s after the start of the test and 30 s before the end of the test.

### 2.4. Statistical Analysis

Data are presented as mean ± standard deviation, unless otherwise stated. Baseline characteristics and hemodynamic variables were compared among groups through a one-way factorial analysis of variance; if a significant difference was detected, intergroup comparisons were performed with Scheffe’s multiple comparison test. A Cox proportional hazard regression analysis was performed to identify independent predictors of cardiac events. Cumulative cardiac event estimates were calculated by the Kaplan–Meier method; differences between the survival curves were assessed by the log-rank test. All analyses were performed with the SPSS 27.0 software package (SPSS Inc., Chicago, IL, USA). A *p*-value of ˂0.05 was considered statistically significant.

## 3. Results

### 3.1. Comparison of Patients with Low, Moderate, and High sUA

A total of 411 patients (mean age ± SD, 81 ± 7 years) were enrolled. Of the 411 patients enrolled, 217 were male (53%). In the total cohort, the mean sUA level was 6.3 mg/dL. We stratified the patients into low- (<5.3 mg/dL), moderate- (5.4–6.9 mg/dL), and high- (>7.0 mg/dL) sUA groups and compared their baseline characteristics (Table 1). There were no significant differences in age or body mass index among the three groups, but there were significantly more male patients in the high-sUA group than in the other groups. Diuretics and sUA-lowering therapy were significantly different among the three groups. LVEF, plasma BNP, and estimated glomerular filtration rate were significantly worse in the high-UA group than in the other groups.

When the patients were stratified whether they used an sUA-lowering agent or not, 96 (23%) were on a sUA-lowering therapy and 315 (77%) were not. Unexpectedly, mean serum sUA level in the patients on an sUA-lowering agent (6.8 mg/dL) was significantly higher (*p* = 0.002) than in those not (6.1 mg/Dl). The sUA-lowering agents prescribed were febuxostat (*n* = 77; 80%), allopurinol (*n* = 18; 19%), and benzbromarone (*n* = 1; 1%).

### 3.2. Comparison of Exercise Capacity and Prognosis in Patients with Low, Moderate, or High sUA Levels

**Figure 1:**
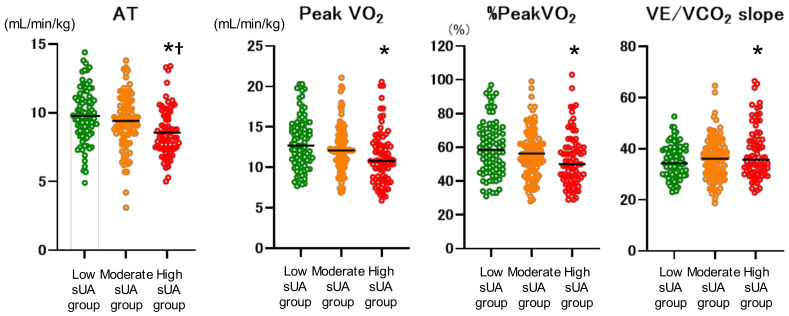
Exercise capacity in patients stratified by serum uric acid (sUA) into three groups (low, <5.3; moderate, 5.4–6.9; and high, >7.0 mg/dL), as assessed by cardiopulmonary exercise testing. AT, anaerobic threshold. * vs. low-sUA group (*p* < 0.05); † vs. moderate-sUA group (*p* < 0.05).

**Figure 2:**
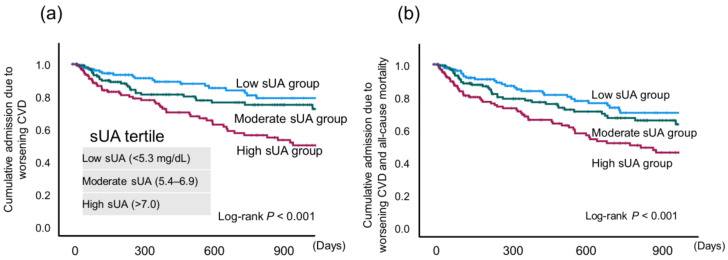
Kaplan–Meier curves for (**a**) cumulative admission due to worsening cardiovascular disease (CVD) and (**b**) cumulative admission due to worsening CVD and all-cause mortality in patients stratified by serum uric acid (sUA) tertile.

Exercise capacity parameters were compared among the low-, moderate-, and high-sUA groups (Figure 1). Anaerobic threshold (AT), peak VO_2_, % peakVO_2_, and VE/VCO_2_ slope were significantly worse in the high-sUA group compared with the low-sUA group. AT was also significantly worse than in the moderate-sUA group. In addition, the high-sUA group showed a significantly higher incidence of the composite endpoint (*p* < 0.001) (Figure 2). 

### 3.3. Cox proportion Hazard Ratio Analysis

A univariate Cox regression analysis, but not a multivariate analysis, indicated that sUA was significantly associated with the composite endpoint (hazard ratio: 1.237; *p* < 0.001) (Table 2). Plasma BNP, serum iron, and albumin levels were significant independent predictors of cardiac event (B = 0.002, −0.013, and −0.075, respectively).

### 3.4. Comparison of the Patients Stratified by Gender

**Figure 3:**
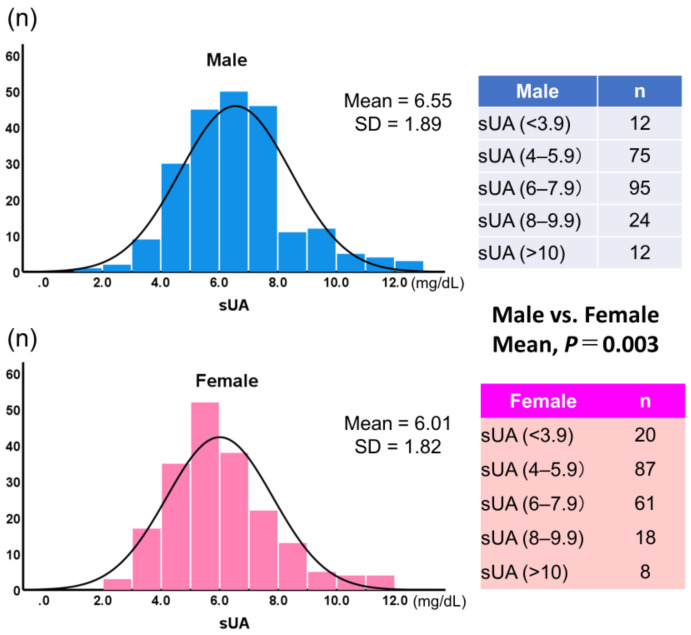
Histograms of serum uric acid (sUA) values by gender. sUA, serum uric acid (mg/dL).

**Figure 4:**
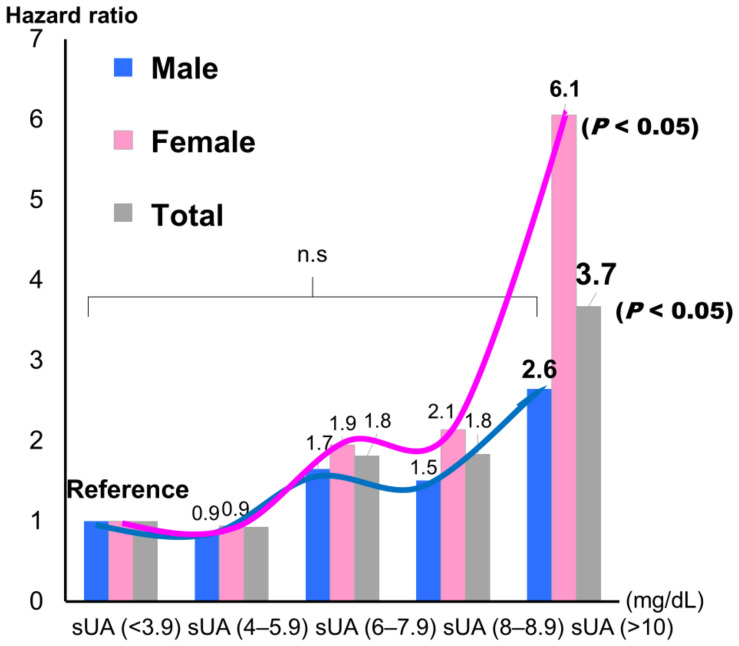
Hazard ratios for the composite endpoint of admission due to worsening cardiovascular disease and all-cause mortality by gender. sUA, serum uric acid (mg/dL).

Figure 3 shows histograms of sUA by gender. Mean sUA was significantly higher in male patients than in female patients (6.55 ± 1.89, 6.00 ± 1.82, respectively; *p* = 0.003). When sUA was classified into five groups and the lowest group was used as the reference, the highest group showed a significantly higher incidence for the composite endpoint, but only in female patients (hazard ratio: 6.12; *p* < 0.05) (Figure 4).

## 4. Discussion

There are four main findings in the present study:

(1) When the patients were divided into three groups according to sUA level (<5.3, 5.4–6.9, and >7.0 mg/dL), the highest sUA group showed a significantly worse exercise capacity and composite endpoint (rehospitalization due to worsening CVD and all-cause mortality; *p* < 0.001) using the Kaplan–Meire method.
(2) Female patients (hazard ratio: 6.12, *p* < 0.05), but not male patients, showed a significantly higher incidence of the composite endpoint following Cox regression analysis.
(3) A univariate Cox regression analysis, but not a multivariate analysis, revealed that sUA was significantly associated with the composite endpoint (hazard ratio: 1.237; *p* < 0.001).

Together, these results suggest that higher sUA is associated with worse exercise capacity and prognosis in older adults with CVD, but this interpretation likely needs to be considered in light of potential gender-associated differences.

### 4.1. Exercise Capacity

In our population, the patients with high sUA (according to tertile) had a significantly lower AT and peak VO_2_, and a significantly higher VE/VCO_2_ slope. In addition, AT had a statistically stronger tendency, in accordance with a previous study [21]. The impact of hyperuricemia on exercise tolerance has rarely been investigated. Leyver et al. [22] evaluated the relationship between sUA concentration and measures of functional capacity obtained through CPX, and they reported an inverse relationship between sUA concentration and peak VO_2_ in heart failure patients. It has also been reported that, in patients with congestive HF, sUA concentration is inversely related to ventilatory AT, independent of the hyperuricemic effects of renal impairment and diuretic therapy [21]. This relationship may be explained by the early switch to anaerobic metabolism and the consequent accumulation of lactate in the cells of heart failure patients. Kinugawa et al. [23] reported that exercise induced the enhanced release of adenosine from the exercising skeletal muscles in HF patients with NYHA III and suggested that the degraded adenosine 5′-monophosphate (AMP) of exercising muscles could be converted into adenosine but not converted into inosine monophosphate (IMP). Although IMP could be converted to AMP through the purine nucleotide cycle, adenosine could be released from skeletal muscle cells due to its membrane permeability, resulting in the loss of adenosine triphosphate in the skeletal muscle and impaired exercise capability. The reduction in the availability of intracellular oxygen caused by the depletion of adenosine triphosphate and accumulation of hypoxanthine and UA may also account for the observed association between AT and sUA. Thus, the relationship between increased sUA concentration and the impairment of exercise tolerance in heart failure could be a consequence of the dysfunction of oxidative metabolism. However, a possible direct role of UA cannot be excluded.

High sUA levels can cause injury to the endothelium by increasing platelet aggregation and through the direct induction of inflammation [13]. UA may also stimulate vascular smooth cell proliferation, but reduce nitric oxide availability [2]. Moreover, high UA levels have been shown to significantly increase angiotensin II expression in cultured endothelial cells, which induces endothelial cell senescence and apoptosis [24]. Finally, elevated sUA levels may lead to endothelial dysfunction, a key feature of chronic HF, which contributes to increased peripheral vasoconstriction and impaired exercise capacity [25].

### 4.2. Prognostic Significance

Of the laboratory parameters examined in the present study, only BNP, serum iron, and albumin were independent predictors of a CV event. These findings are consistent with previous reports of the associations between these independent predictors and the pathophysiology of CVD in older adults [26,27,28]. Furthermore, sUA did not show a significant association with CV events in a multivariate analysis. In addition, in both men and women, when the patients were stratified into five sUA groups and the lowest sUA group (<3.9 mg/dL) was set as the reference group, the hazard ratio slightly decreased for the second-lowest group, although not significantly so, and then increased with each subsequent group (Figure 4). 

The pathophysiologic role of UA has been studied in a wide variety of disease processes and debated for decades; however, a complete understanding is still not at hand [1,2,3]. Most previous studies have focused on the pathophysiologic effects of high levels of UA. More recently, research has also shifted to the impact of hypouricemia, with multiple studies showing the potentially damaging effects that can be caused by abnormally low levels of sUA. Thus, both high and low sUA concentrations may be risk factors for renal, CV, and pulmonary comorbidities, and may have a U-shaped association with CV mortality [29,30]. To confirm these issues, further research is needed.

### 4.3. Gender Differences

sUA concentrations are usually higher in males than in females [31]. In addition, vascular parameters are usually higher in males than in females [31]. Due to a gender difference in the relationship between sUA and Framingham Risk Score, sUA can only be used as marker of risk of future CVD events in males [32]. Gender differences in CVD pathophysiology, clinical presentation, prevention, and management have also been observed [33,34]. Lifestyle may play an important role in the difference in the development of CVDs between genders [33]. In accordance with previous studies [31,35], we found gender differences in the older adults with CVD examined in the present study. However, the hazard ratio for CV events was larger in female patients than in male patients. An sUA concentration of over 10 mg/dL, especially in female patients, indicated a high risk of experiencing a CV event. Recently, a growing body of evidence has suggested that elevated sUA levels may increase the risk of cardiovascular morbidity and mortality in CVD [36,37]. Aggressive sUA-lowering therapy may be beneficial to CV outcomes [38], but more research is needed. However, the optimal management of all comorbidities is of the utmost importance in older adults with CVD, irrespective of whether aggressive sUA-lowering therapy is used.

### 4.4. Clinical Implications

Laboratory parameters are commonly evaluated in daily practice because they can be determined through inexpensive, repeatable, and noninvasive testing. Here, we did not include specialized parameters relevant to frailty, such as plasma interleukin 6 and tumor necrosis factor alpha concentrations, as we wished to test only those biomarkers used in general practice. To our knowledge, this is the first study to investigate the prognostic significance of sUA and exercise capacity in older adults hospitalized for worsening CVD, and to find a gender difference among these patients. The primary goals of CVD therapy are to improve quality of life and extend survival. The assessment of sUA in daily clinical practice might be useful for diagnostic tests to determine exercise capacity or prognosis even in older adults with CVD.

### 4.5. Study Limitations

This was a single-center study with a small sample size. Moreover, we did not assess repeated measures over time in the enrolled patients. We did not check alcohol consumption or assess changes in the trajectory of exercise capacity or frailty due to medical intervention or cardiac rehabilitation. The population had various conditions, including chronic coronary syndrome, heart failure, aortic stenosis, and peripheral artery disease. All these groups were very different in terms of exercise capacity and they took different therapeutic drugs. The patients were hospitalized for different reasons, which determined the heterogeneity of the population. Older patients are more likely to be under-therapeutically optimized, so their symptoms could be due to this more than to UA. Finally, the findings of our study, based on a non-randomized design, largely generate hypotheses and call for similar analyses of larger and more recent databases, prospective follow-up studies, and confirmation through randomized clinical trials.

## 5. Conclusions

A high sUA was significantly associated with worse exercise capacity in a CPX of older adults with CVD. Although sUA might have some degree of prognostic significance in this population, this significance may differ between men and women.

## Figures and Tables

**Table 1:** Baseline characteristics of the patients stratified by serum uric acid (sUA) level.

	Low sUA (≤5.3 mg/dL) (*n* = 141)	Moderate sUA (5.4–6.9 mg/dL) (*n* = 141)	High sUA (≥7.0 mg/dL) (*n* = 129)
Age (y)	81 ± 7	81 ± 7	81 ± 8
Female (%)	58	45	37
Body mass index (kg/m^2^)	21.9 ± 3.6	22.3 ± 3.6	22.2 ± 3.9
Hypertension *n*, (%)	93 (66)	89 (63)	76 (59)
Diabetes mellitus, *n*, (%)	26 (28)	37 (26)	30 (23)
Hyperlipidemia, *n*, (%)	58 (41)	56 (40)	43 (33)
***Underlying diseases***			
Heart failure, *n*, (%)	101 (72)	104 (74)	107 (83)
Cardiomyopathy, *n*	7	9	14
Ischemic, *n*	17	17	17
Hypertensive, *n*	10	17	12
Tachycardia, *n*	24	32	27
Valvular disease, *n*	19	12	19
Bradycardia, *n*	15	11	11
Other, *n*	9	6	7
AMI, *n*, (%)	8 (7)	13 (11)	7 (6)
Angina pectoris, *n*, (%)	22 (18)	17 (14)	10 (9)
Other, *n*, (%)	10 (8)	7 (6)	5 (5)
***Medications***			
Diuretic (%)	38	50	67
Tolvaptan (%)	13	17	24
ACE-I/ARB (%)	38	44	42
Beta blocker (%)	41	54	47
Antiplatelet (%)	48	40	33
Anticoagulant (%)	35	39	39
Spironolactone (%)	48	40	33
sUA-lowering (%)	17	22	32
LVEF (%)	59.5 ± 10.1	56.1 ± 12.9	52.3 ± 15.4 *†
E/e′	15.3 ± 6.0	14.8 ± 5.9	15.8 ± 6.7
BNP (pg/dL)	111 ± 123	179 ± 248 *	200 ± 205 *
eGFR	60.8 ± 17.6	52.4 ± 25.5 *	44.8 ± 16.1 †
GNRI	98.4 ± 10.7	98.8 ± 11.5	97.1 ± 11.4
AT (mL/min/kg)	9.6 ± 2.0	9.6 ± 3.0	8.6 ± 1.9 *†
Peak VO_2_ (mL/min/kg)	12.9 ± 3.2	12.3 ± 3.1	11.2 ± 3.3 *
% peakVO_2_ (%)	59.0 ± 16.1	56.7 ± 14.2	52.8 ± 15.7 *
VE/VCO_2_ slope	35.4 ± 8.0	36.1 ± 8.4	40.0 ± 8.5 *

* vs. low sUA (*p* < 0.05); † vs. moderate sUA (*p* < 0.05). AMI, acute myocardial infarction; AT, anaerobic threshold; BNP, B-type natriuretic peptide; E/e′, ratio of transmitral Doppler early filling velocity to tissue Doppler early diastolic mitral annular velocity; eGFR, estimated glomerular filtration rate; GNRI, Geriatric Nutritional Risk Index; UA, uric acid; VE/VCO_2_, the minute ventilation/carbon dioxide production.

**Table 2:** Univariate and multivariate Cox regression analysis for composite endpoint.

Univariate	B	Wald	*p*	Exp(B)	95% CI	
					Lower	Upper
**Uric acid**	0.213	22.604	<0.001	1.237	1.133	1.350
**Multivariate**	**B**	**Wald**	***p***	**Exp(B)**	**95 CI**	
					**Lower**	**Upper**
**BNP**	0.002	15.844	<0.001	1.002	1.001	1.004
**Fe**	−0.013	7.742	0.005	0.987	0.977	0.996
**Albumin**	−0.775	6.616	0.010	0.461	0.255	0.832
Creatinine	0.735	2.472	0.116	2.085	0.834	5.211
Total cholesterol	−0.006	2.359	0.125	0.994	0.986	1.002
Uric acid	0.105	1.978	0.160	1.110	0.960	1.284
Triglyceride	0.003	1.223	0.269	1.003	0.998	1.007
C-reactive protein	−0.055	0.385	0.535	0.946	0.795	1.127
Blood urea nitrogen	0.008	0.155	0.694	1.008	0.971	1.046
eGFR	0.002	0.034	0.854	1.002	0.980	1.024

BNP, B-type natriuretic peptide; eGFR, estimated glomerular filtration rate. Bold parameters show the statistical significance (*p* < 0.05).

## Data Availability

Data are contained within the article.
